# Monodispersed Sirolimus-Loaded PLGA Microspheres with a Controlled Degree of Drug–Polymer Phase Separation for Drug-Coated Implantable Medical Devices and Subcutaneous Injection

**DOI:** 10.1021/acsabm.2c00319

**Published:** 2022-07-16

**Authors:** Zilin Zhang, Ekanem E. Ekanem, Mitsutoshi Nakajima, Guido Bolognesi, Goran T. Vladisavljević

**Affiliations:** † Department of Chemical Engineering, Loughborough University, Loughborough LE11 3TU, U.K.; ‡ Guangxi Key Laboratory of Green Chemical Materials and Safety Technology, Beibu Gulf University, Qinzhou 535011, China; § Department of Chemical Engineering, University of Bath, Bath BA2 7AY, U.K.; ∥ Faculty of Life and Environmental Sciences, University of Tsukuba, 1-1-1 Tennoudai, Tsukuba, Ibaraki 305-8572, Japan

**Keywords:** step microfluidic emulsification, drug delivery, drug-eluting medical devices, biodegradable polymer, poly(lactic-*co*-glycolic acid), controlled drug release

## Abstract

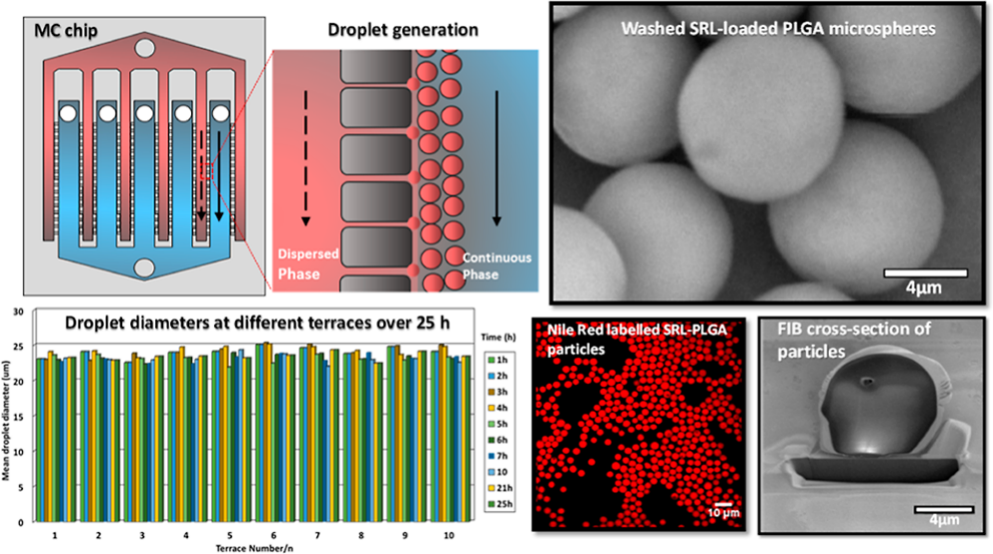

Monodispersed sirolimus (SRL)-loaded poly(lactic-*co*-glycolic acid) microspheres with a diameter of 1.8, 3.8, and 8.5 μm were produced by high-throughput microfluidic step emulsification—solvent evaporation using single crystal silicon chips consisted of 540–1710 terraced microchannels with a depth of 2, 4, or 5 μm arranged in 10 parallel arrays. Uniform sized droplets were generated over 25 h across all channels. Nearly 15% of the total drug was released by the initial burst release during an accelerated drug release testing performed at 37 °C using a hydrotropic solution containing 5.8 M *N*,*N*-diethylnicotinamide. After 24 h, 71% of the drug was still entrapped in the particles. The internal morphology of microspheres was investigated by fluorescence microscopy using Nile red as a selective fluorescent stain with higher binding affinity toward SRL. By increasing the drug loading from 33 to 50 wt %, the particle morphology evolved from homogeneous microspheres, in which the drug and polymer were perfectly mixed, to patchy particles, with amorphous drug patches embedded within a polymer matrix to anisotropic patchy Janus particles. Janus particles with fully segregated drug and polymer regions were achieved by pre-saturating the aqueous phase with the organic solvent, which decreased the rate of solvent evaporation and allowed enough time for complete phase separation. This approach to manufacturing drug-loaded monodisperse microparticles can enable the development of more effective implantable drug-delivery devices and improved methods for subcutaneous drug administration, which can lead to better therapeutic treatments.

## Introduction

An implantable medical device is any instrument used for diagnostic and/or therapeutic purposes such as a stent or balloon, which is introduced into the human body by clinical intervention and intended to remain in place for an extended period.^1^ Although these devices are subject to rigorous health and safety requirements, their intimate and prolonged contact with the human body increases the risks of adverse events and reactions, such as formation of the bacterial biofilm^2^ and restenosis.^3^ Restenosis is re-narrowing of the artery previously treated for blockage and represents the greatest risk factor limiting the success of percutaneous coronary interventions.^4^

Coronary balloons and stents can be coated with an anti-restenotic drug which is slowly released at the contact area between the device and the vessel wall to inhibit neointimal hyperplasia, the main cause of restenosis.^5,6^ Paclitaxel and sirolimus (SRL) are the two major anti-restenotic drugs in drug-eluting medical devices.^7^ Paclitaxel diffuses more readily through the plaque and the vessel wall, but it is less effective in suppressing restenosis and has a narrower therapeutic window compared to SRL.^8^ As a result, a growing number of SRL-eluting stents^9^ and balloons^10^ are receiving clinical approval.

One of the most important inventions in drug-eluting technology is the adoption of a drug carrier (excipient) to facilitate drug transfer to the vessel wall.^11^ Without the excipient, the drug forms crystalline lumps on the device surface, which inhibits drug transfer, especially in drug-coated balloons due to short contact time between the inflated balloon and the artery wall (30–120 s). Other benefits of encapsulating drugs include enhanced adhesion of the drug to the device surface, increased stability of the coating during handling, its improved adherence to the vessel wall, and extended drug release to prolong therapeutic effects and minimize side effects.^11^ Coating for drug-eluting medical devices often consists of a homogeneous excipient layer saturated with a particular drug. This approach provides high initial drug levels, but the drug concentration rapidly decreases because the drug diffuses quickly out of the surface region of the coating, and the coating soon becomes depleted of the drug. One way of achieving a uniform drug release over prolonged time is to prepare microspheres composed of a drug-excipient mixture and then disperse these micro-reservoirs uniformly in a thin layer of another hydrophobic material.^12^ After implantation, drug molecules are slowly released from embedded micro-reservoirs to the secondary hydrophobic matrix and then diffuse through the matrix layer to the artery wall. The secondary matrix should adhere well to the artery wall and should provide a good dispersion of micro-reservoirs. The compounds that can be used to form a secondary matrix are sterols, phospholipids, fats, and hydrophobic surfactants.^12^

Poly(lactic-*co*-glycolic acid) (PLGA) is the most widely used biomaterial for drug encapsulation,^13^ generally recognized as safe (GRAS) by FDA and the European Medicines Agency (EMA).^14^ Currently, there are 19 FDA-approved PLGA-based drug products on the market, mainly in the form of microspheres.^15^ The particle size is a key factor in their design, as it affects the drug encapsulation efficiency, product injectability, distribution in the body, and drug release rate.^14,16^ Conventional methods of producing PLGA microspheres, such as spray drying,^17^ coacervation, high-speed mixing, or high-pressure homogenization combined with solvent evaporation,^18^ result in wide particle size distributions with a coefficient of variation (CV) of particle sizes of 30–50%. The particle size uniformity can be improved through expensive classification processes, but they are associated with high drug and polymer losses. Better control over the particle size can be achieved using membrane emulsification, but the CVs are still relatively high, between 7 and 20%.^19^

Recent advances in microengineering and semiconductor technologies have enabled fabrication of microfluidic chips for generation of monodisperse droplets with CVs less than 3%.^20^ Droplet microfluidics offers many advantages over conventional emulsification methods including unprecedented control over the droplet size with no polymer–drug losses, high drug encapsulation efficiency because droplets are formed at negligible shear, and operation in a closed environment enabling sterile manufacturing to meet the Current Good Manufacturing Practice (CGMP) regulations.

The main problem of conventional microfluidic devices such as T-and ψ-junctions^21,22^ is a low droplet productivity because droplets are produced one at a time, and the maximum throughput is limited by dripping to jetting transition.^23^ Scaling up T-junctions and flow focusing nozzles is challenging because droplet formation in these geometries is controlled by the shear rate at the interface between the dispersed phase and CP, which is sensitive to fluid flow rates. As a result, small flow rate fluctuations can result in large droplet size variations, making parallelization of these devices difficult.^24^ Step microfluidic emulsification is an alternative approach of generating uniform droplets in a low shear environment based on exploiting a sudden change in the channel geometry from shallow channels to a deep and wide microwell.^25,26^ Although the mechanism was discovered in the mid-1990s,^27^ it was largely ignored until recent years.^28,29^ The droplet size in step emulsification devices can be tuned solely by the geometry of shallow channels. The effect of fluid flow rates is negligible, which allows for easy multiplication of individual channels.^30,31^

Here, we report for the first time the production of monodispersed SRL-loaded PLGA microparticles by step microfluidic emulsification. The microparticles are fabricated with the tunable size and internal morphology using single-crystal silicon chips consisting of multiple arrays of grooved MCs. The fabricated particles can be used for subcutaneous SRL administration or as drug micro-reservoirs in SRL-coated balloons, thereby offering new opportunities for the development of improved restenosis treatments based on either subcutaneous drug injections or implantable drug delivery systems.

## Materials and Methods

### Materials

SRL or rapamycin (SRL, purity > 99%) was purchased from Chunghwa Chemical Synthesis & Biotech Co. Ltd (Taiwan). PLGA (*M*_w_ = 10,000 g mol^–1^) containing 75% dl-lactic acid and 25% glycolic acid, purchased from Wako Pure Chemical Industries (Osaka, Japan), was used as an excipient. Poly(vinyl alcohol) (PVA, *M*_w_ = 13,000–23,000 g mol^–1^, 87–89% hydrolyzed, Sigma-Aldrich, UK) dissolved in pure Milli-Q water or dichloromethane (DCM)-saturated Milli-Q water served as a water-soluble surfactant. DCM (HPLC grade, Fisher Scientific, UK) and isopropyl acetate (IPAc, Sigma-Aldrich, UK) were used as solvents for PLGA and SRL. Nile red (Sigma-Aldrich, UK) was added to the dispersed phase as a fluorescent dye to investigate the internal morphology of the particles.

### MC Chips

**Figure 1:**
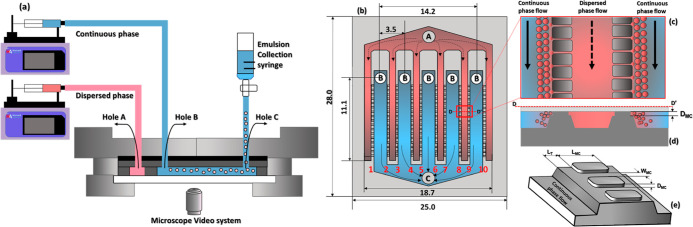
Schematics of the microfluidics rig: (a) stainless steel module with two syringe pumps, a collection syringe, and a reflected-light microscope for observation of droplet generation; (b) silicon chip with 5 cross-flow channels and 10 parallel rows of terraced microchannels (MCs) (red labels are terrace numbers): A = inlet hole for the dispersed phase, B = inlet holes for the continuous phase (CP), and C = outlet hole for the emulsion. Red regions are dead-end channels for the dispersed phase, and blue regions are cross-flow channels for the CP; (c) magnified top view of a single dead-end channel in section D–D; (d) magnified side view of the dead-end channel with two terrace walls; (e) terrace wall with two MCs on the top and a deep well at each side.

Three cross-flow silicon microchips with terraced MCs arranged in 10 parallel arrays were used for droplet generation (Figure 1b). The MC dimensions are shown in Table 1. The chips are designed in Nakajima Lab (NFRI, NARO, Tsukuba, Japan) and EP Tech Co. Ltd., commercialized by EP Tech, Co., Ltd., and microfabricated by photolithography and deep reactive ion etching by Hitachi Power Semiconductor Device, Ltd., Hitachi, Japan.

**Table 1:** Geometry of the Chips Used in This Study (See Also Figure 1e): *D*_MC_ = MC Depth; *W*_MC_ = MC Width; *d*_h_ = Hydraulic Diameter of a MC; *L*_MC_ = MC Length; *L*_T_ = Terrace Length; *N*_MC_ = Total Number of MCs; *n*_MC_ = Number of MCs in a Single Row

CHIP	CMS6-1	CMS6-2	CMS6-3
*D*_MC_ (μm)	5	4	2
*W*_MC_ (μm)	18	8	12
*d*_h_ (μm)	7.0	4.1	3.3
*L*_MC_ (μm)	140	140	140
*L*_T_ (μm)	60	30	30
*N*_MC_	540	1850	1710
*n*_MC_	54	185	171

### Chip Cleaning

The used chip was washed in a Fisherbrand FB11003 ultrasound bath at 10% power using a mild detergent dissolved in a mixture of water and ethanol. The chip was then rinsed with Milli-Q water, oven-dried, and transferred to a plasma cleaner (Fischione 1020) to oxidize persistent organic contaminants that could not be removed by the detergent. The plasma oxidation was performed at 0.01 Pa for 15 min (pure O_2_) or 20 min (25% O_2_ and 75% Ar). After cleaning, the chip was placed on a watch glass and stored in the CP. To evaluate the cleaning process, the contact angle between a water droplet and a dry chip surface was measured by using the Krüss Model DSA 100 Advanced drop shape analyzer.

### Drop Generation

A clean chip was placed in a module (Figure 1a) filled with the CP. The dispersed phase was then delivered to the module via hole A (Figure 1a–d) and distributed across six dead-end channels until it overflowed the upstream terraces. The flow rate was then adjusted to 0.02–0.05 mL/h (Table 2) to force the dispersed phase through MCs. The CP was delivered through hole B (Figure 1b) and flowed through five cross-flow channels. When disk-like dispersed phase jets on the downstream terraces reached the terrace edge, they were pulled into the CP stream by the imbalance in the capillary pressure along the interface, which caused the jet to pinch-off and release drops. The emulsion was removed through hole C. The bottom window was used for the observation of droplet generation by using a reflected-light microscope (MS-511-M; Seiwa Optical Co., Ltd., Tokyo).

**Table 2:** Experimental Conditions, Emulsion Formulations, and Droplet/Particle Sizes for Different Sample Runs

		S1	S2	S3	S4	S5	S6	S7	S8	S9
nominal channel depth, *D*_MC_ (μm)		5	4	2	4	4	4	4	4	4
dispersed phase composition (wt %)	SRL	1.32	1.32	1.32	1.32	1.64	1.76	1.80	1.84	2.25
	PLGA	2.68	2.68	2.68	2.68	2.34	2.24	2.20	2.16	2.25
	IPAc	72.00	72.00	72.00	72.00	72.00	72.00	72.00	72.00	71.63
	DCM	24.00	24.00	24.00	24.00	24.00	24.00	24.00	24.00	23.88
CP composition (wt %)	PVA	1.50	1.50	1.50	1.50	1.50	1.00	1.00	1.00	1.00
	Milli-Q water	98.50	98.50	98.50	98.50	98.50	99.00	99.00	99.00	99.00
drug loading (wt %)		33	33	33	33	42	44	45	46	50
total solids in the dispersed phase (wt %)		4	4	4	4	4	4	4	4	5
droplet production time		48 h	24 h	24 h	24 h	48 h	24 h	24 h	24 h	24 h
flowrate (mL/h)	dispersed phase	0.15	0.03	0.01	0.03	0.05	0.02	0.05	0.02	
	CP	1.00	1.00	1.00	1.00	1.00	1.00	1.00	1.00	1.00
mean droplet size (μm)		23.5	13.8	5.2	13.8	13.8	13.8	13.6	13.8	13.5
CV of droplet sizes (%)		3.0	2.5	3.5	2.5	3.0	2.6	2.0	2.5	2.4
predicted particle size (μm)		7.0	4.1	1.5	4.1	4.2	4.2	4.3	4.3	4.5
mean particle size (μm)		8.5	3.8	1.8	4.2	4.0	4.2	4.2	4.2	4.3
CV of particle sizes (%)		4.5	5.2	6.0	5.2	4.1	4.3	5.1	4.8	4.6

### Droplet Size Analysis

Droplet generation was observed using a color CCD camera (LCL-211H, Watec America Corp., USA), 30 frames per second, 720 × 480 resolution, and recorded with Ulead Video Studio 11 SE DVD video editing software (InterVideo Digital Technology Corp.). The CV was calculated as
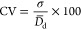
1where σ is the standard deviation, and *D̅*_d_ is the average droplet diameter based on at least 30 droplets. The droplet diameters were measured using ImageJ software. The particle diameter was predicted using the mass balance of non-volatile solids in the dispersed phase by assuming zero particle porosity
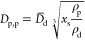
2where *D*_p,p_ is the predicted particle diameter, *x*_s_ is the mass fraction of non-volatile solids in the dispersed phase, and ρ_p_ and ρ_d_ are the density of particles and droplets, respectively.

### Particle Preparation

The particles were formed upon DCM evaporation from the droplets. The residual PVA was removed from the suspension through multiple washing cycles. Each washing cycle consisted of centrifuging the suspension at 3500 rpm for 10 min, removing the supernatant with a Pasteur pipette, adding an equal volume of 0.05 wt % Tween 20 solution, and vortexing for 5–10 s. The concentration of residual PVA in the supernatant was measured using the PerkinElmer Lambda 35 UV–vis spectrometer. After final wash, the level of the supernatant in the vial was reduced just above the level of the sediment and the particles were dried using the AdVantage 2.0 bench top freeze dryer. The vial content was first frozen to −20 °C and then left to dry at 16 Pa using a condenser temperature of −86 °C.

### Particle Characterization

#### Confocal Laser Scanning Microscopy

The particle morphology was visualized using the Bio-Rad RAD200 confocal laser scanning system mounted on the Nikon Eclipse TE300 inverted microscope and connected to a computer running Zeiss LaserSharp 2000 software. A suspension of dried particles was placed on a microscope slide and excited with an argon laser at 488 nm and helium-neon laser at 543 nm. To enhance the observation of the polymer–drug distribution, the total emission was divided into two images that were captured by using two separate photomultiplier tubes (PMTs): PMT1 captured fluorescence at 515 ± 30 nm (green region) and PMT2 captured fluorescence above 570 nm (yellow-red region).

#### Scanning Electron Microscopy

Benchtop scanning electron microscopy (SEM) (model TM3030, Hitachi) was utilized to investigate the surface morphology of the particles and the efficiency of the washing process. All micrographs were taken with an aperture size of 30 μm using a beam current of 2.1 nA and a voltage of 10 kV. The washing was considered successful if no PVA crystals could be found on SEM images and no PVA film bridges between the particles. Particles with a smooth clean surface and no sign of particle–particle bridges were considered properly washed.

#### Focused Ion Beam-Scanning Electron Microscopy

This imaging was carried out using a dual-beam focused ion beam-scanning electron microscope (FIB-SEM) instrument (Nova 600 NanoLab, FEI Company, Hillsboro, Oregon, USA), which combines ultra-high-resolution SEM and precise FIB etching and deposition. The FIB acceleration voltage was 30 kV, and the ion beam current was 20 nA. The cross section was cleaned at 7 nA with the final cleaning at 3 nA. To preserve the exposed cross-section surface during imaging at 2 min/image, the current was reduced to 30 pA.

#### X-ray Diffraction Spectroscopy

X-ray diffraction spectroscopy (XRD) patterns of raw SRL, blank PLGA particles, raw PLGA, and SRL-loaded PLGA particles (1:1 SRL/PLGA blend) were recorded using a Bruker D2 Phaser diffractometer equipped with a one-dimensional LYNXEYE detector. The samples were exposed to Cu Kα (1.54184 Å) radiation (30 kV, 10 mA) passed through a 0.5 mm thick nickel filter over the 2θ range from 2 to 40°, with a step size of 0.02° and a rotation speed of 15 rpm. The spectra were obtained using Bruker’s proprietary EVA 2.0 software. Triple runs were performed for each sample for reproducibility.

#### Attenuated Total Reflection–Fourier Transform Infrared Spectroscopy

Attenuated total reflection–Fourier transform infrared (ATR–FTIR) analysis was carried out using the Thermo Fisher Scientific Nicolet iS50 FTIR spectrometer. Between 2 and 3 mg of the powdered sample was placed onto the universal diamond ATR top-plate, and the spectrum was recorded within 32 s over the range of 4000–400 cm^–1^.

### Drug Release Study

A 100 mL of dissolution medium was prepared in a volumetric flask by mixing 10 mL of 20 vol % Tween 20 solution, 10 mL of absolute ethanol (200 proof), 25.9 mL of 5.8 M *N*,*N*-diethylnicotinamide (DENA), 44.1 mL of Milli-Q water, and 10 mL of 10× phosphate-buffered saline at 37 °C for 1 h. To measure the SRL release profile, 2 mg of freeze-dried particles was placed into a microcentrifuge tube, and 2 mL of the dissolution medium was added. The tube was capped, alternately sonicated, and vortex-agitated for 2 min to break apart any particle aggregates and then placed in an agitator and incubated at 37 °C and 250 rpm for 25 min. After that, the tube was centrifuged for 5 min at 13,000 rpm. The supernatant was extracted from the sample using a syringe and transferred into a labeled high performance liquid chromatography (HPLC) vial. The removed supernatant was replenished with fresh dissolution medium, and the same procedure was repeated for the samples collected after 1, 2, 3, 4, 5, 7, and 24 h. After 24 h, the remaining sample was quenched by adding 2 mL of acetonitrile (ACN) to fully dissolve drug-deprived particles and release the remaining drug. All samples were stored at −20 °C before analysis. The concentration of SRL in the samples was determined by HPLC (Agilent Technologies, 1100 series, Hewlett Packard) using the procedure described elsewhere,^32^ based on the calibration line shown in Figure S4.

## Results and Discussion

### Mean Droplet/Particle Size

Table 2 summarizes the droplet/particle sizes and their CVs for each run. The mean droplet diameter (*D̅*_d_) in the dripping regime was varied from 5.2 to 13.8 to 23.5 μm by changing the depth of MCs, *D*_MC_, from 2 to 4 to 5 μm (samples S1–S3). The ratio of *D̅*_d_ to the hydraulic channel diameter (*d*_h_) was 3.3–3.4 for *D*_MC_ = 4 and 5 μm, which is close to the droplet size to pore size ratio in SPG membrane emulsification.^33^ For grooved MCs with trapezoidal cross section and no terrace (*L*_T_ = 0), *D̅*_d_/*d*_h_ was around 3.0.^34^ For *L*_T_ > 0, *D̅*_d_/*d*_h_ ranged from just above 3 to 6, which is the maximum value that can be achieved only for very long terraces. In this study, *D̅*_d_/*d*_h_ values were within this range at *D*_MC_ = 4 and 5 μm.

The mean particle size achieved in the chip with a channel depth of 5, 4, and 2 μm was 8.5, 3.8, and 1.8 μm, respectively (samples S1–S3). As a comparison, all commercial injectable, long-acting PLGA-based depot formulations have a mean particle size of at least 8 μm, such as Lupron Depot (∼8 μm),^35^ Sandostatin LAR (∼50 μm),^36^ Vivitrol (40–60 μm),^37^ and Ozurdex (∼300 μm). These medicines are injected either subcutaneously or intramuscularly using relatively large needle diameters (19–23 gauge), due to broad particle-size distributions, for example, the volume median diameter of the 1-month Lupron Depot microspheres produced using the conventional emulsification—spray drying technique is (11.4 ± 0.5) μm, but 10 vol % of the particles are bigger than 30 μm, and the maximum particle size is 100 μm.^38^ These PLGA microspheres are injected through a 23G needle with an inner diameter of 116 μm. PLGA particles that could be administered using thinner needles would make clinical use more patient-friendly because a reduced needle size leads to reduced pain.^39^ In this work, the mean particle size in sample S1 was 8.5 μm, but 95% of the particle sizes were in a very narrow range of 7.8–9.2 μm. The minimum particle size in drug-eluting balloon (DEB) coatings is about 1.5 μm, which corresponds to a drug release half-life of about 14 days under physiological conditions. Smaller particle sizes do not provide sufficiently extended drug release due to an increased surface area to volume ratio and reduced diffusional pathway for the drug.^12^ The maximum particle size in DEB coatings is approximately the size of a red blood cell, 6–8 μm,^12^ to prevent embolization of capillaries due to any release of the particles into the blood stream during or after treatment.

### Sustained Droplet Generation

**Figure 2:**
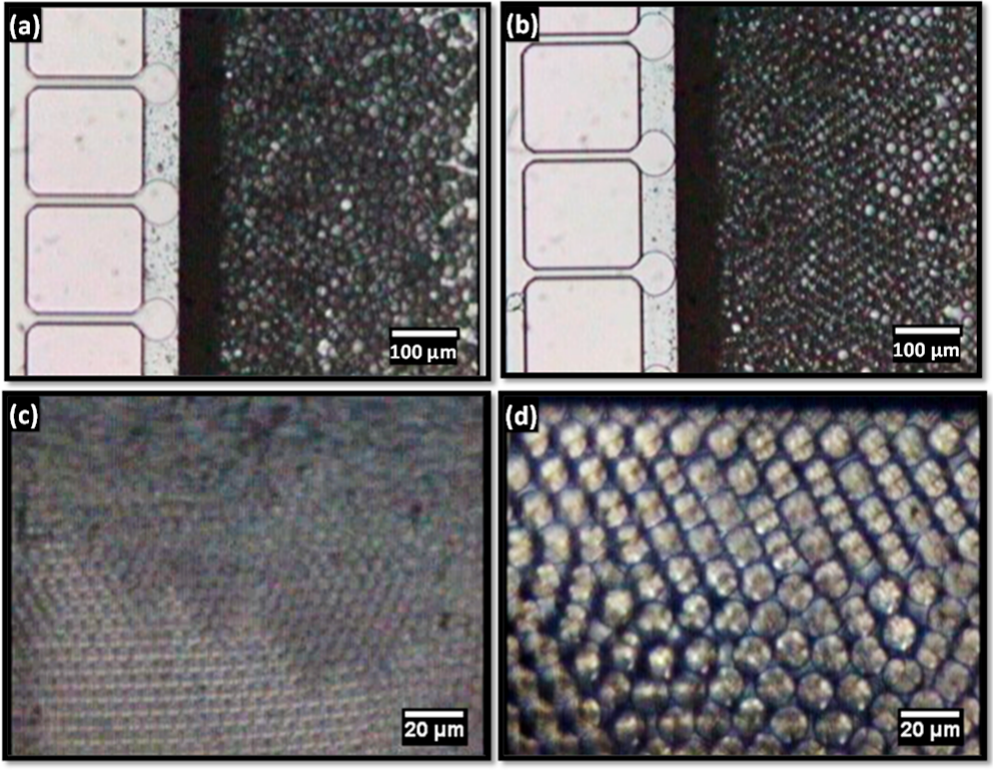
Droplet generation in different chips: (a) CMS6-1 chip after 1 h of continuous operation; (b) CMS6-1 chip after 24 h of continuous operation; (c) CMS6-3 chip after 2 h of continuous operation; (d) CMS6-2 chip after 2 h of continuous operation. Samples S1–S3 in Table 2.

During droplet generation, the dispersed phase expands on the terrace into discs, as shown in Figure 2a, due to the confinement in the vertical direction. The Laplace pressure of the dispersed phase on the terrace is *p*_1_ = γ(1/*r*_1_ + 2/*D*_MC_), where γ is the interfacial tension and *r*_1_ is the disc radius, as shown in Figure S2 in the Supporting Information. Confined droplets expand on the terrace due to influx of the dispersed phase fluid, but *p*_1_ does not change because *D*_MC_ ≪ *r*_1_ and *p*_1_ ≈ 2γ/*D*_MC_, that is, the Laplace pressure on the terrace does not depend on the disc radius *r*_1_ but only on the terrace depth. When the dispersed phase reaches the terrace edge and enters a well, it is no longer confined in the orthogonal direction and expands in all directions. The Laplace pressure in the well is *p*_2_ = γ/*r*_2_, where *r*_2_ is the droplet radius in the well, which is much greater than *D*_MC_ and thus *p*_1_ ≫ *p*_2_. The difference in the Laplace pressure (*p*_1_–*p*_2_) induces a sudden flow of the dispersed phase into the well, and the dispersed phase jet breaks via Rayleigh–Plateau instability.^34^Video S1 in the Supporting Information shows that droplet generation through the Laplace pressure induced a snap-off for sample S1. A confined droplet expands on the terrace, and once it reaches the terrace edge, the interface is quickly pulled into the well, where it releases a droplet and then retraces back. This pinch-off mechanism requires no shear force and occurs entirely due to a variable curvature of the interface, which affects the Laplace pressure.^40^ We have proved this feature by varying the CP flow rate in the dripping regime, but it had no impact on the droplet size. The droplet size was unaffected even when the CP flow rate was very low (Video S2) or equal to zero, which caused the formed droplets to accumulate under the cover glass and self-assemble into densely packed single-layer or multi-layer hexagonal arrays, as shown in Figure 2c,d.

Cleaning of the chip using the described procedure was very effective and led to sustained droplet generation for at least 24 h. The change in the contact angle of water during cleaning a silicon surface is shown in Figure S1 in the Supporting Information. The contact angle of a Milli-Q water drop sitting on a contaminated silicon surface was ∼60° (Figure S1a). After washing the chip with DCM, the contact angle was reduced to 32.4° (Figure S1b) due to partial removal of organic contaminants. After complete removal of the persistent organic layer by plasma oxidation, the contact angle was reduced to 16.9° (Figure S1c), which means that the silicon surface was highly hydrophilic.

Droplet generation pictures in the CMS6-1 chip after continuous operation for 1 and 24 h are compared in Figure 2a,b. Although the wall wettability and droplet shapes on the terrace were slightly altered after 24 h due to prolonged contact of the silicon surface with emulsion ingredients (DCM, SRL, and PVA), no significant change in the droplet size was observed. Any wetting of the terrace walls by the dispersed phase reduces the Laplace pressure on the terrace (*p*_1_), and its radius of curvature becomes greater than *D*_MC_/2. In the limiting case, for a wetting angle of 90°, the radius of curvature approaches infinity, and the Laplace pressure gradient tends to zero. This leads to negligible driving force for droplet pinch-off and causes a continuous outflow of the dispersed phase from the channels and formation of big polydisperse droplets.^41^

**Figure 3:**
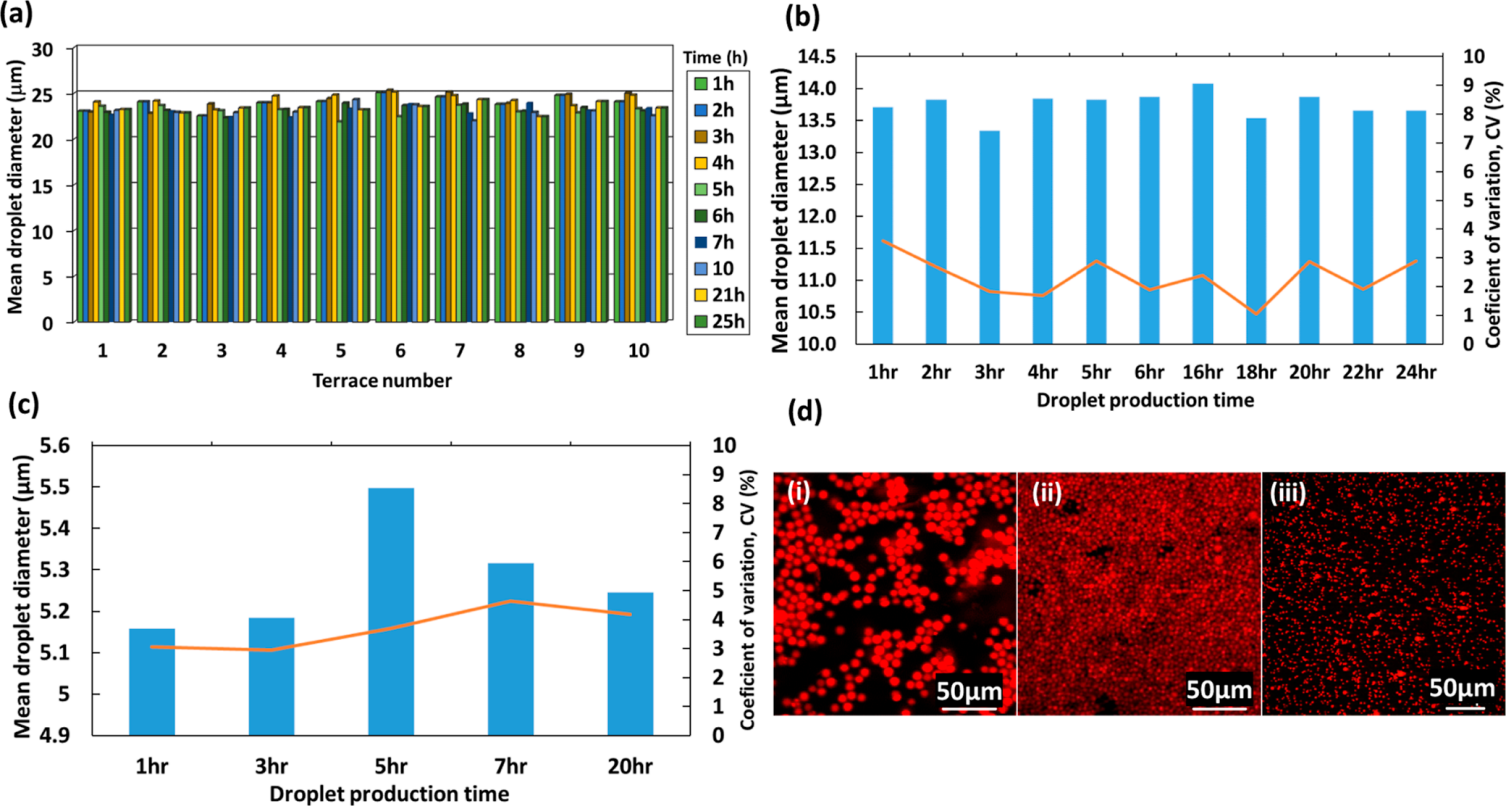
(a) Mean droplet diameter in the CMS6-1 chip at different terraces over 25 h; (b) mean droplet diameter (bars) and CV (solid line) in the CMS6-2 chip over 24 h; (c) mean droplet dimeter (bars) and CV (solid line) in the CMS6-3 chip over 20 h; (d) confocal laser scanning microscopy (CLSM) images of SRL-loaded PLGA particles fabricated in runs S1 (i), S2 (ii), and S3 (iii) in Table 2.

Long-term droplet production stability in the CMS6-2 chip was compared for samples S2 and S5 and is shown in Table 2. As can be seen, the mean droplet size after 24 and 48 h was the same (13.8 μm), but the CV increased slightly from 2.5% after 24 h to 3% after 48 h. The mean droplet size in different chips was measured for extended periods of time with the results shown in Figure 3. The temporal fluctuations of droplet sizes from different terraces in the CMS6-1 chip are shown in Figure 3a. The droplet size variations between different terraces were below 1.5 μm, and a mean droplet size of 23.5 μm was stable over time. In addition, 89–95% of all MCs in the chip was actively producing droplets at any time, and all channels were active at a certain time during the investigated period.

The droplet generation regime shifts from the dripping regime (small and uniform drops) to the continuous outflow regime (large and non-uniform drops) at the critical dispersed-phase velocity, which depends on channel geometry, interfacial tension, and the viscosity ratio between the two phases.^42^ When triolein droplets were generated in a 1 wt % sodium dodecyl sulfate solution, the critical velocity in grooved channels with the similar geometry was 2.2 mm/s.^41^ In the S1 experiment, the average velocity in MCs was 4.2 mm/s, but the dripping regime still existed. It can be explained by the fact that DCM is less viscous than triolein, and the critical velocity is higher at lower dispersed-phase viscosity.

Similar studies were carried out in CMS6-2 and CMS6-3 chips, and the results are shown in Figure 3b,c, respectively. Due to their smaller channels, droplet sizes generated by these chips were smaller than those in the CMS6-1 chip. For the CMS6-2 chip, the 90% confidence interval of the mean droplet size was (13.8 ± 0.6) μm with CV = 2.5%. According to the National Institute of Standards and Technology, particles are monodispersed if at least 90% of the particles have a size within 5% of the median size, *D̅*_d_. For normal size distribution, 90% of the particle sizes is within 1.64 standard deviations (σ) of the mean. Thus, droplets are monodispersed if 1.64σ ≤ 0.05*D̅*_d_ or CV ≤ 3%. Therefore, the 90% confidence limits of the mean for monodisperse droplets should be (13.8 ± 0.7) μm. In the CMS6-3 chip, the 90% confidence interval of the mean droplet size was (5.2 ± 0.3) μm with a CV of 3.5%. The fact that CV > 3% can be attributed to the larger error in measuring the size of smaller droplets.

### Particle Washing

The final product will be contaminated with PVA if particles are not washed. PVA is GRAS by the FDA. Also, the European Food Safety Authority (EFSA) has approved the use of PVA as a food additive if the total intake of PVA does not exceed 4.8 mg/kg bw/day. However, residual PVA can affect bulk properties of the product and should be removed. In the first place, the PVA concentration in the CP should be minimized in order to reduce the initial level of PVA contamination and the number of washing cycles required. It was found that the PVA concentration in the aqueous phase could be reduced from 1.5 to 1 wt % without noticeable impact on the mean droplet size and CV value (S5 and S6 samples in Table 2). The reduction in the PVA concentration below 1 wt % had a negative impact on the droplet size uniformity (the data are not shown here).

**Figure 4:**
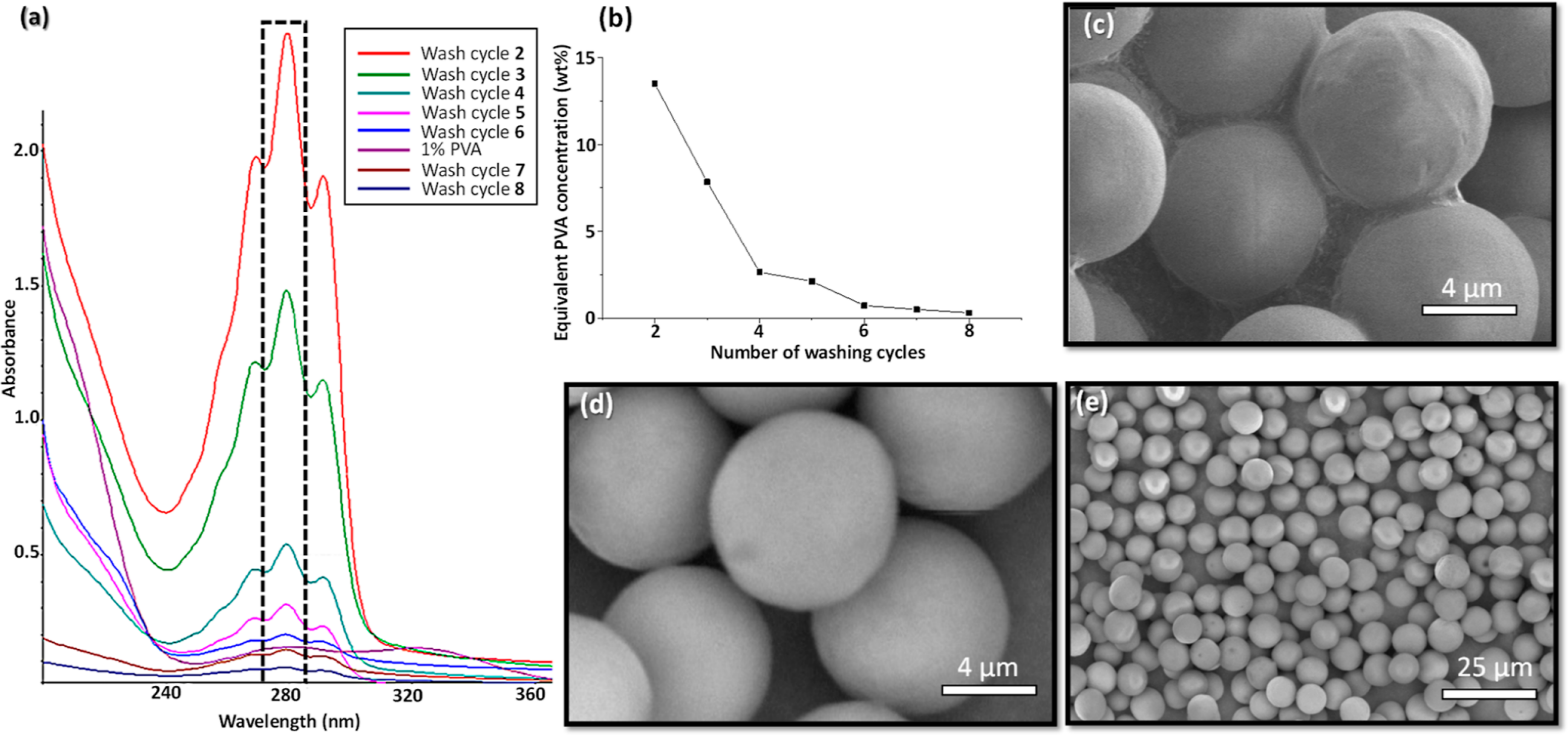
Effect of the number of washing cycles on the purity of the supernatant and particle morphology for the sample S2: (a) UV–visible absorption spectra of the supernatant after each washing cycle and for 1 wt % PVA solution; (b) equivalent PVA concentration of the supernatant as a function of the number of washing cycles (the PVA concentrations in the plot are estimated from the heights of the UV–vis peak at 280 nm based on the assumption that the supernatant is a pure PVA solution); (c) SEM image of SRL-loaded PLGA particles without washing; and (d,e) SEM images of the same sample after eight washes.

Hence, the washing procedure was investigated using sample S1 but the PVA concentration in the CP was reduced from 1.5 to 1 wt %. We have tried to quantify the amount of PVA in the supernatant by UV–vis spectroscopy by measuring the height of the absorbance peak at 280 nm, which was reduced with an increase in the number of washing cycles. The UV–vis analysis of standard solutions of PVA in the concentration range of 0.5–5 wt % revealed that the height of the adsorption peak at 280 nm was proportional to the PVA concentration in the solution (Figure S3 in the Supporting Information). Although the UV–vis spectra clearly show that the supernatant was increasingly less turbid, the method could not be used to quantify the concentration of residual PVA in the supernatant because the supernatant peak after 2nd, 3rd, 4th, and 5th wash was much higher than the peak for pure 1 wt % PVA solution, as shown in Figure 4a,b. It was probably caused due to interference from tiny particle debris in the supernatant, released during washing, that can absorb more UV light than dissolved PVA molecules. The peak at 280 nm almost completely disappeared after 6 cycles. SEM imaging was carried out to confirm the effectiveness of the washing procedure, as shown in Figure 4c–e. As shown in Figure 4c, unwashed particles were aggregated due to binding by the PVA film deposited between dried particles. The formation of particle clusters through PVA bridges can alter all particle size-dependent properties of the product, such as syringe ability, dispersibility in the secondary polymer matrix, and drug release rate. After eight washes, the particle surface was clean and smooth, without any visible PVA film, as shown in Figure 4d,e. However, a small fraction of PVA probably remains on the particle surface because PVA forms an interconnected network with PLGA,^43^ which cannot be washed away from the surface. The amount of molecularly bound PVA is too small to be quantified, and it does not affect the particle size distribution. However, the particle interactions with cell membranes in the body can be affected due to a different chemistry of the surface. The amount of residual PVA adsorbed on the particle surface depends on the miscibility of the organic solvent with water. A higher amount of adsorbed PVA can be expected in the particles prepared from the solvent blend with a higher amount of IPAc due to higher solubility of IPAc in water compared to DCM.

### Solvent Evaporation and Freeze Drying

The size and morphology of drug-loaded particles depend on the droplet size, emulsion formulation, and solvent evaporation rate. The solvent evaporation rate can be controlled by adjusting the temperature and the degree of pre-saturation of the CP with the organic solvent. Two different batches of sample S1 were produced using PVA solutions with different degrees of IPAc saturation (Table 3).

**Table 3:** Mean Particle Size before and after Freeze Drying^a^

	before freeze drying		after freeze drying	
sample	mean size (μm)	CV (%)	mean size (μm)	CV (%)
S1A	7.5	5.3	6.7	4.3
S1B	9.6	6.2	8.1	5.0

a The CP was 1.5 wt % PVA saturated with IPAc (S1A) or pure 1.5 wt % PVA solution (S1B).

**Figure 5:**
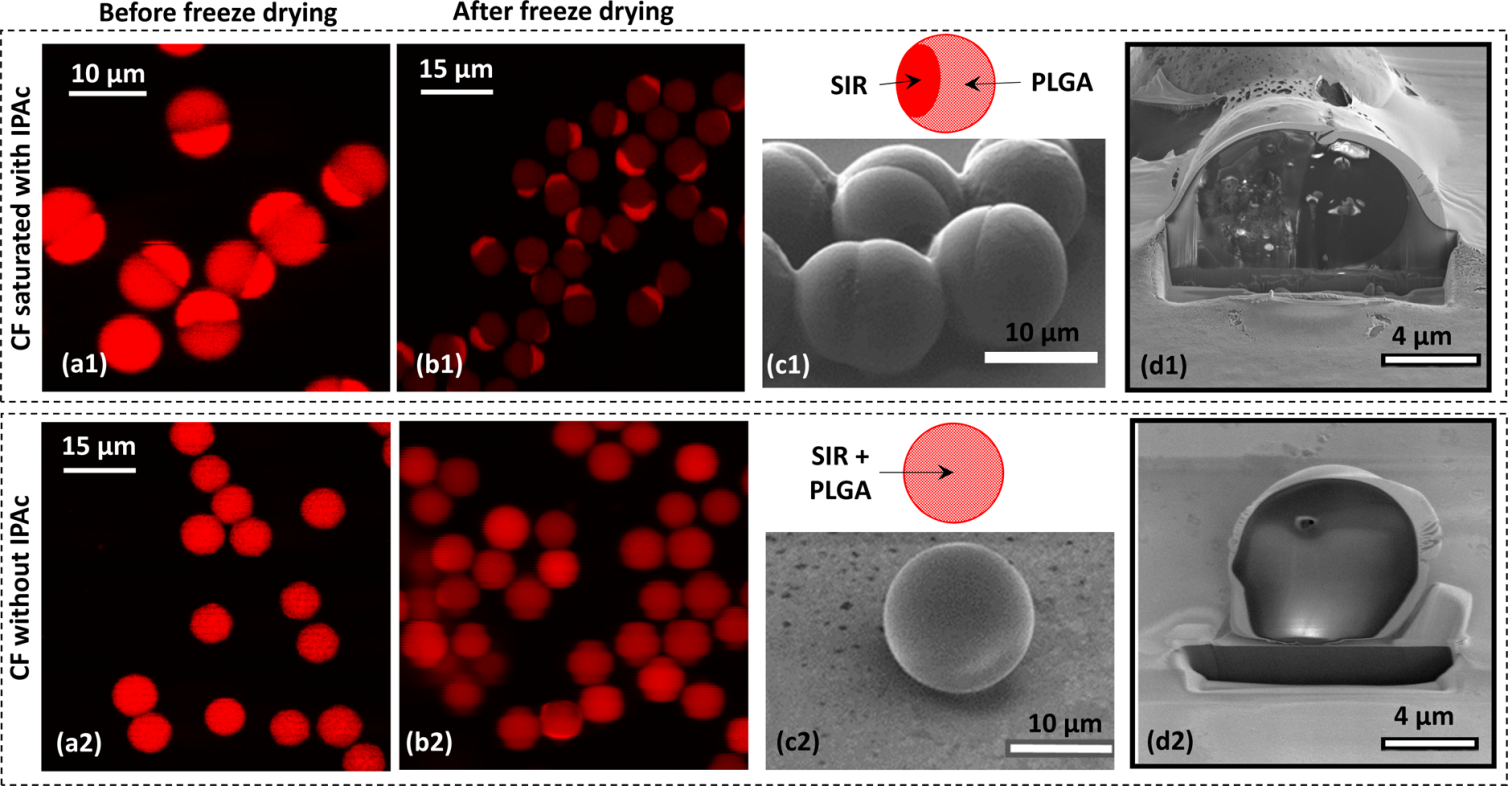
Effect of saturating the CP with the organic solvent on the particle morphology. (a1,a2): CLSM images of particles before freeze drying; (b1,b2): CLSM images of particles after freeze drying; (c1,c2): SEM images of intact particles and the schematic view of their internal structure; (d1,d2): SEM images of particles cross-sectioned by FIB. The CP was 1.5 wt % PVA solution saturated by IPAc in (a1–d1) and pure 1.5 wt % PVA solution, in (a2–d2). In (a1,b1), the brighter particle parts are rich in SRL and the darker parts are rich in PLGA. The drug loading in all particles was 33 wt %.

For batch A, the CP was 1.5 wt % PVA solution pre-saturated with IPAc, while for batch B the CP was pure 1.5 wt % PVA solution, thereby enabling higher rates of solvent extraction from the droplets. In both cases, the initial droplet size was 22 μm with a CV of 4.7%, but larger particles were formed by faster solvent removal, as shown in S1B batch in Table 3. The particle size in both batches was above 7 μm, which is the theoretical prediction from the mass balance equation based on a completely non-porous solid matrix. The total specific volume of a solid polymer is the sum of the volume occupied by the polymeric chains and the free volume of the material. If an organic solvent is rapidly removed from the droplets, the result will be the formation of excess free volume trapped due to rapid solvent removal that can be reduced either slowly through physical aging or more rapidly through freeze drying. The particle size in both samples shrank by 10–15% following freeze drying and was close to the size predicted by eq 2. As can be seen in Figure 5, SRL was more uniformly distributed when the CP was a pure PVA solution, as shown in Figure 5a2–d2, probably due to faster solvent removal and insufficient time for drug–polymer phase separation. When the CP was pre-saturated with IPAc, a phase boundary between the drug and the polymer could be observed, as shown in Figure 5a1–d1.

Initially, SRL and PLGA are fully mixed within the newly formed droplets due to the entropic gain (Δ*S*_M_ > 0), as a result of their random distribution in the organic phase and favorable solvent-PLGA and solvent-SRL interactions (Δ*H*_M_ < 0), resulting in a negative Gibbs free energy of mixing, Δ*G*_M_ < 0, where Δ*G*_M_ = Δ*H*_M_ – *T*Δ*S*_M_. As both solvents are removed, the concentration of SRL and PLGA increases and the entropic gain becomes insufficient to overcome the enthalpic penalty (Δ*H*_M_ > 0) due to disfavored PLGA–SRL interactions, which are more pronounced in more concentrated solutions. As a result, a homogeneous organic phase is separated into polymer-rich and drug-rich domains in the process of spinodal decomposition. Further solvent removal causes segregation of SRL-rich domains and formation of Janus particles with a single crescent-moon-shaped SRL-rich region on one side of the particle, as shown in Figure 5c1. This step is possible only if the solvent is removed sufficiently slowly. Nile red is a hydrophobic compound whose fluorescence is stronger in less polar environments.^44^ SRL-rich regions are less polar than PLGA-rich regions, and therefore, they exhibit a higher fluorescence intensity with brighter red color as shown in Figure 5b1. Since the drug loading was 33%, a SRL-rich region occupies smaller particle volume than a PLGA-rich region. The phase separation is incomplete, and both regions contain both components. Indeed, as shown in the FIB-SEM image in Figure 5d1, drug patches are embedded within the PLGA region, while PLGA patches are incorporated within the SRL region.

An observation that faster solvent removal from droplets leads to incomplete segregation of SRL and PLGA and more uniform drug distribution in the polymer matrix is consistent with the results of our previous study, where composite PCL–PLA particles were produced by emulsification—solvent evaporation using the dispersed phase composed of a mixture of ethyl acetate (partially soluble in water) and DCM (insoluble in water). More uniform polymer distribution in the particles was observed when the dispersed phase contained more ethyl acetate.^45^

### Effect of Drug Loading

**Figure 6:**
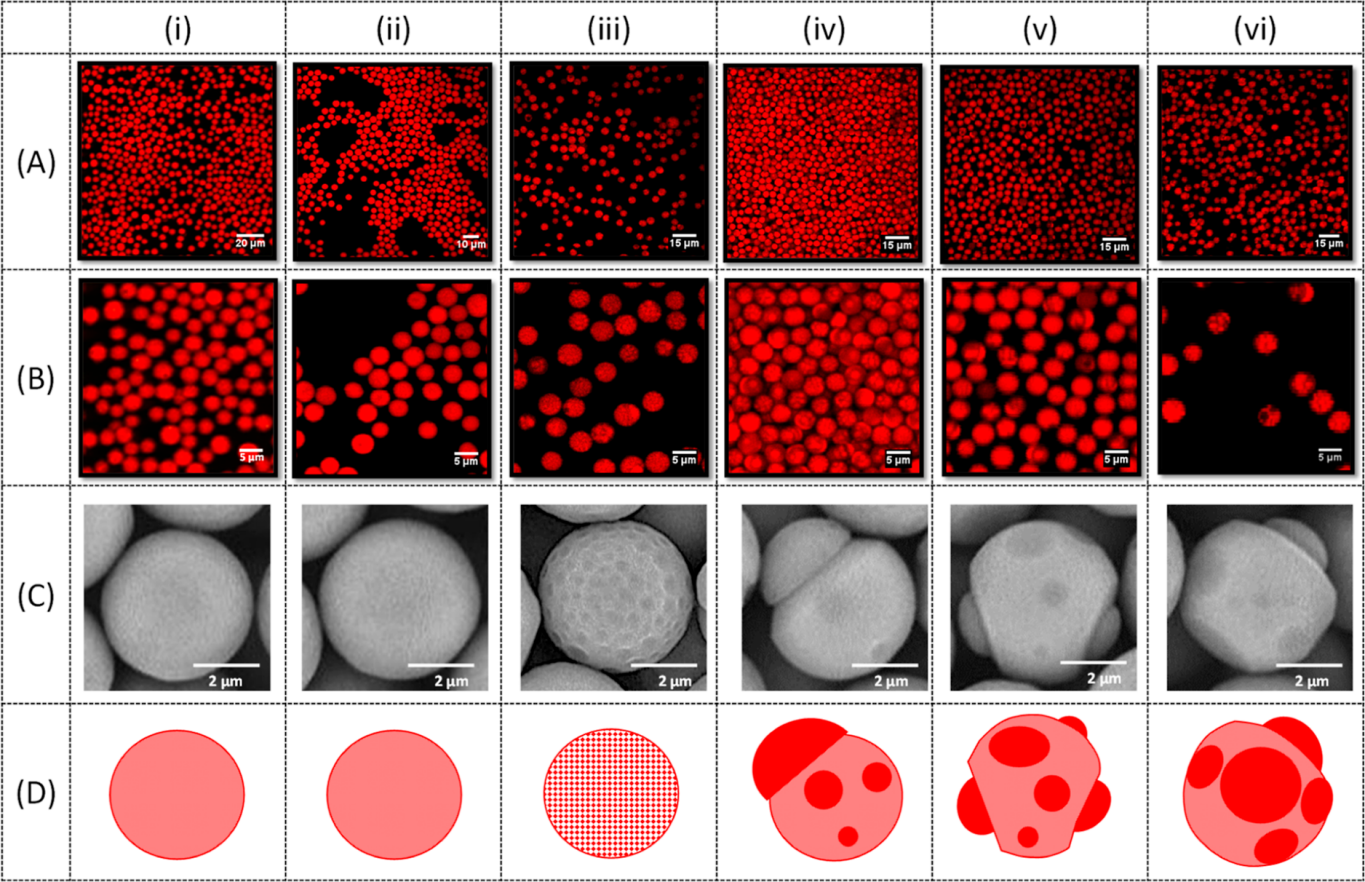
Effect of drug loading on the particle morphology. The drug loading was: (i) 33 wt % (S4); (ii) 42 wt % (S5); (iii) 44 wt % (S6); (iv) 45 wt % (S7); (v) 46 wt % (S8); and (vi) 50 wt % (S9). The experimental conditions and emulsion formulations are shown in Table 2. The fluorescence microscopy images are taken at 20× (A) and 60× (B) magnification. The SEM images of the particles are shown in (C), and the schemes of particle morphologies are shown in (D).

The effect of drug loading on the particle morphology is shown in Figure 6. The extent of drug–polymer phase separation depended on the drug loading and ranged from a uniform drug distribution for the drug loadings of 33 and 42 wt %, as shown in Figure 6(i,ii), to small uniform SRL patches embedded within a PLGA matrix for a drug loading of 44 wt %, as shown in Figure 6(iii), to patchy Janus particles for the drug loading from 46 to 50 wt %, as shown in Figure 6(iv–vi). Homogeneous particles are preferred since they are more resistant to attrition during the coating process due to their smooth surface and regular spherical shape with no internal interfaces that can cause particle breakage. Apart from the drug loading in the particles, the amount of drug in the coating can be tailored by the particle concentration in the coating suspension, which can vary between 10 and 65 wt % to provide the amount of SRL on the expandable portion of the catheter surface from 1 to 10 μg/mm.^12^

Patchy and patchy Janus particles are not stable during storage in aqueous solutions and slowly evolve into fully separated Janus morphology due to Ostwald ripening. In this process, drug from small patches is dissolved into the surrounding aqueous phase and deposited onto larger patches. The process also occurs at a later stage of the solvent evaporation process through internal diffusion of SRL facilitated by the residual solvent. The similar patch coarsening process occurred in PCL–PLA composite particles leading to various particle morphologies.^46^

### Microparticle Characterization

**Figure 7:**
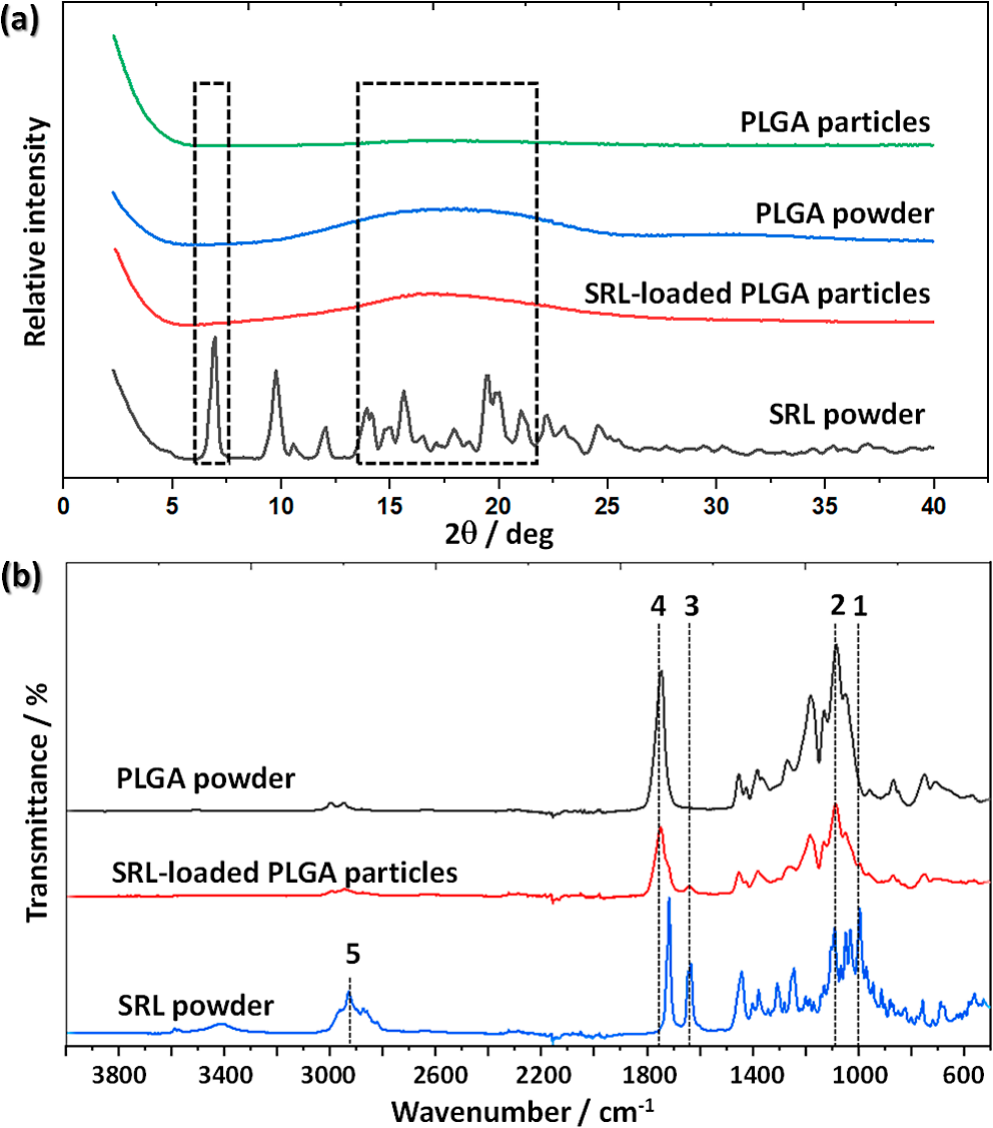
(a) XRD patterns of raw SRL powder, raw PLGA powder, SRL-loaded PLGA particles (sample S4), and blank PLGA particles; (b) ATR–FTIR spectra of raw SRL powder, raw PLGA powder, and SRL-loaded particles (sample S4).

XRD analysis was carried out on freeze-dried S4 microparticles to reveal the presence of any impurities in the sample and assess its crystallinity. The XRD patterns of raw SRL powder, raw PLGA powder, blank PLGA particles produced from the dispersed phase containing 4 wt % PLGA and SRL-loaded PLGA particles with 33% drug loading produced from the dispersed phase containing 4 wt % total solids (sample S4) are shown in Figure 7a. A series of sharp and well-defined SRL peaks at 2θ values between 7 and 25° reveal a crystalline structure of SRL and can be attributed to X-ray diffraction from different lattice planes in SRL crystallites. A large amorphous hump between 2θ values of 8 and 26° was observed for raw PLGA powder due to the absence of regular atomic structures in the amorphous polymer. A very shallow and broad peak of blank PLGA particles indicates that these particles are more amorphous than raw PLGA powder, probably due to random supramolecular rearrangement of PLGA chains following solvent evaporation. However, a more prominent peak of SRL-loaded PLGA particles compared to blank PLGA particles indicates their more crystalline structure owing to the drug entrapment within a polymer matrix. Therefore, encapsulation of SRL within PLGA can be confirmed by XRD analysis. The absence of sharp peaks in the XRD pattern of SRL-loaded PLGA particles indicates that SRL was either molecularly dispersed in PLGA or present in the form of amorphous domains.

Figure 7b shows FTIR spectra for pure PLGA, pure drug, and drug-loaded PLGA particles. Observed IR absorption peaks reveal the presence of characteristic bonds in PLGA and SRL. For example, the peaks at ∼1000 (1) and 1640 cm^–1^ (3) for SRL were due to the out-of-plane C–H bending vibrations in −CH=CH– bonds and the C=C stretching vibrations, respectively. These two bonds do not exist in PLGA, and consequently, no peaks were found in the PLGA spectrum at these frequencies. On the other hand, strong peaks at ∼1100 (2) and 1750 cm^–1^ (4) for PLGA occurred due to the C–O–C and C=O stretching vibrations in ester groups, respectively. In addition, the peak at ∼3400 cm^–1^ (5) for SRL was due to the O–H stretching vibrations. In PLGA macromolecules, the O–H bonds are present only in terminal groups. Also, Figure 7b shows that SRL was incorporated uniformly within the particles rather than being separated onto the surface of the particles since the peaks (1), (3), and (5) are negligible for SRL-loaded PLGA particles.

### In Vitro Drug Release Study

**Figure 8:**
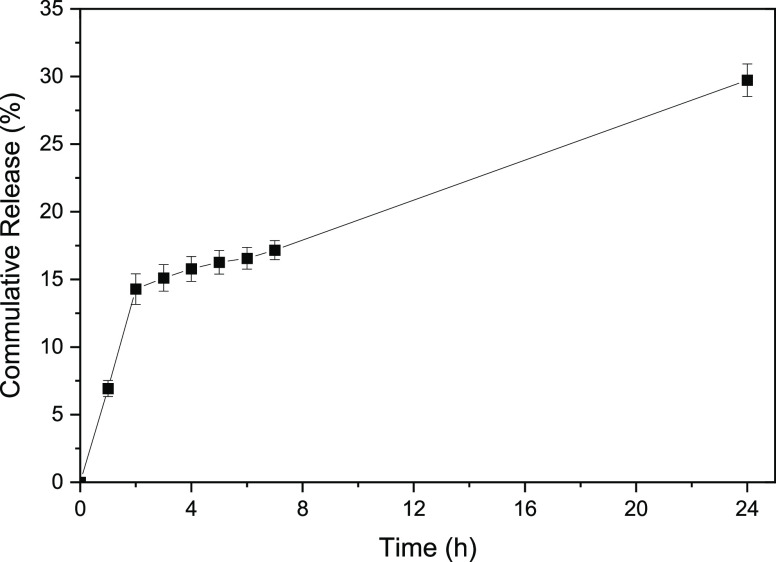
Percentage of SRL released from PLGA particles as a function of time under accelerated conditions for two replicate determinations on sample S3 (Table 1). After 24 h, all particles in the sample were dissolved with ACN.

The percentage of drug released over 24 h from PLGA particles with a size of 5.2 μm loaded with 33 wt % SRL is shown in Figure 8. No difference in release kinetics between two replicates was observed, indicating a high batch-to-batch consistency of the release profile due to consistent particle size and structure. The duration of drug release in injectable, clinical, long-acting PLGA-based depot formulations typically ranges from 1 week to 6 months.^15^ In the case of DEB applications, since the resolution of inflammation in the region of balloon dilation typically takes 4–12 weeks, it is desirable to provide therapeutic tissue levels of the drug for about 4 to 12 weeks following the treatment.^12^ To reduce the duration of drug release during in vitro testing, as shown in Figure 8, the drug release rate was enhanced by adding DENA, which acts as an effective hydrotrope (non-micellar solubilizer) of poorly water-soluble drugs. For example, the aqueous solubility of paclitaxel from PLGA particles was increased by several orders of magnitude in the presence of DENA.^47^ It has been explained either by faster degradation of PLGA in the presence of DENA^47^ due to hydrolysis of ester bonds in PLGA chains^48^ or by higher drug solubility due to DENA’s non-stoichiometric accumulation around the drug.^49^

Most long-acting PLGA-based depot formulations exhibit an initial burst release which often releases a quarter of the total drug on the first day. As a result, the drug concentration on the first day of administration is often 100 times higher than the steady state drug concentration in the blood.^15^ In this study, nearly 15% of encapsulated drug was released within the first 2 h by the initial burst release. The cause of the initial burst release is still not fully understood, but it can be attributed to the diffusion of drug molecules from the surface layer of the microparticles. As shown in Figure 8, the initial burst release was followed by zero-order kinetics (the constant release rate) from 2 to 24 h, during which the cumulative amount of the released drug reached 29%. After 24 h, 71% of the drug was still entrapped in the particles and was released by quenching the particles with ACN. In the steady-state release period, the drug depletion from the particles was counterbalanced by an increased permeability of the particle matrix due to DENA-induced PLGA degradation leading to a constant release rate of SRL. Increased permeability of the polymer matrix is a result of the decrease in the average molecular weight of the polymer due to PLGA hydrolysis and reduced degree of polymer chain entanglements. Thus, the mobility of PLGA chains increases, leading to increased matrix permeability to SRL, according to the free volume theory of diffusion.^48^

## Conclusions

Monodispersed SRL-loaded PLGA microspheres of controlled size and internal morphology were successfully produced by microfluidic step emulsification and subsequent solvent evaporation. A stable droplet generation in the dripping regime was maintained for 48 h and resulted in droplets of highly consistent size created across all MC terraces. The droplet size in the dripping regime was controlled by the smallest dimension of droplet forming MC, confirming that droplet pinch-off was driven by the gradient of the capillary pressure established when a deformed droplet squeezed in a shallow MC enters a deep microfluidic well.

The size of the generated microspheres was accurately predicted from the dispersed phase formulation and the size of the parent droplets. The microspheres were small enough and highly monodispersed to be safely injected in the blood stream using hypodermic needles of any size and can be used as drug delivery coatings for implantable medical devices, such as cardiac stents and balloon catheters.

The particle morphology has a profound effect on the drug release rate^50,51^ with less significant burst release expected to occur from microspheres with a more homogeneous internal structure. In this study, the degree of drug–polymer phase separation was controlled by varying the drug to polymer ratio in the microspheres and the rate of solvent removal. Suppressed solvent evaporation and higher drug loadings led to more pronounced polymer–drug phase separation, resulting in heterogeneous patchy, patchy Janus, and Janus particle morphologies.

The developed accelerated drug release testing method allowed us to release nearly 30% of SRL within 24 h, with the initial bust release consuming nearly a quarter of the drug initially present in the microspheres.

To conclude, monodispersed SRL-loaded PLGA microspheres generated by step microfluidic emulsification/solvent evaporation can open up new routes for the manufacturing of more effective implantable devices and for the development of improved subcutaneous drug injection methods for restenosis treatment. More broadly, the proposed microfluidic approach for the reliable, robust, and scalable manufacturing of highly-monodisperse drug-loaded microparticles could be extended to other excipient/drug formulations, hence improving the performance of many other subcutaneous drug treatments and implantable devices for controlled drug release.

In the next phase of this study, the fabricated microspheres will be dispersed in a hydrocarbon or fluorocarbon solution to form the coating formulation that will be applied to the surface of a balloon catheter and let dry to form the coating. After that, the coated device will be used to assess the drug transfer to an arterial vessel.

## Supporting Information Available

S1. Contact angle measurements between pure water and the silicon chip surface; S2. Droplet formation by step microfluidic emulsification using a terraced microchannel; S3. Calibration curve of PVA for UV−vis analysis; S4. calibration curve of SRL for HPLC analysis (PDF)
Video S1: Droplet generation through the Laplace pressure difference (MP4)
Video S2: Monodispersed droplets accumulated in a cross-flow channel at low continuous phase flow rate (MP4)

## Abbreviations

CLSM: confocal laser scanning microscopy
SEM: scanning electron microscope
DCM: dichloromethane
SRL: sirolimus
PLGA: poly(lactic-*co*-glycolic acid)
PVA: poly(vinyl alcohol)
XRD: X-ray diffractometry
ATR–FTIR: attenuated total reflection–Fourier transform infrared
MCE: microchannel emulsification
UV–vis: ultraviolet–visible and visible spectrophotometers

## References

[ref1] Joung Y.-H. Development of Implantable Medical Devices: From an Engineering Perspective. Int. Neurourol. J. 2013, 17, 98–106. 10.5213/inj.2013.17.3.98. 24143287 PMC3797898

[ref2] Alvarez-Lorenzo C.; Concheiro A. Smart Drug Release from Medical Devices. J. Pharmacol. Exp. Ther. 2019, 370, 544–554. 10.1124/jpet.119.257220. 30967402

[ref3] Buccheri D.; Piraino D.; Andolina G.; Cortese B. Understanding and Managing In-Stent Restenosis: A Review of Clinical Data, from Pathogenesis to Treatment. J. Thorac. Dis. 2016, 8, E1150–E1162. 10.21037/jtd.2016.10.93. 27867580 PMC5107494

[ref4] Munro E.; Chan P.; Patel M.; Betteridge L.; Gallagher K.; Schachter M.; Sever P.; Wolfe J. Consistent Responses of the Human Vascular Smooth Muscle Cell in Culture: Implications for Restenosis. J. Vasc. Surg. 1994, 20, 482–487. 10.1016/0741-5214(94)90149-x. 8084043

[ref5] Puranik A. S.; Dawson E. R.; Peppas N. A. Recent Advances in Drug Eluting Stents. Int. J. Pharm. 2013, 441, 665–679. 10.1016/j.ijpharm.2012.10.029. 23117022 PMC3567608

[ref6] Htay T.; Liu M. W. Drug-eluting stent: a review and update. Vasc. Health Risk Manag. 2005, 1, 263–276. 10.2147/vhrm.2005.1.4.263. 17315599 PMC1993957

[ref7] Ma X.; Oyamada S.; Gao F.; Wu T.; Robich M. P.; Wu H.; Wang X.; Buchholz B.; McCarthy S.; Gu Z.; Bianchi C. F.; Sellke F. W.; Laham R. Paclitaxel/Sirolimus Combination Coated Drug-Eluting Stent: In Vitro and in Vivo Drug Release Studies. J. Pharm. Biomed. Anal. 2011, 54, 807–811. 10.1016/j.jpba.2010.10.027. 21126843 PMC3008332

[ref8] Pires N. M. M.; Eefting D.; De Vries M. R.; Quax P. H. A.; Jukema J. W. Sirolimus and Paclitaxel Provoke Different Vascular Pathological Responses after Local Delivery in a Murine Model for Restenosis on Underlying Atherosclerotic Arteries. Heart 2007, 93, 922–927. 10.1136/hrt.2006.102244. 17449502 PMC1994420

[ref9] Abizaid A. Sirolimus-Eluting Coronary Stents: A Review. Vasc. Health Risk Manag. 2007, 3, 191–201. 10.2147/vhrm.2007.3.2.191. 17580729 PMC1994032

[ref10] Cortese B.; di Palma G.; Latini R. A.; Elwany M.; Orrego P. S.; Seregni R. G. Immediate and Short-Term Performance of a Novel Sirolimus-Coated Balloon during Complex Percutaneous Coronary Interventions. The FAtebenefratelli SIrolimus COated-Balloon (FASICO) Registry. Cardiovasc. Revascularization Med. 2017, 18, 487–491. 10.1016/j.carrev.2017.03.025. 28365415

[ref11] Turner E.; Erwin M.; Atigh M.; Christians U.; Saul J. M.; Yazdani S. K. In Vitro and in Vivo Assessment of Keratose as a Novel Excipient of Paclitaxel Coated Balloons. Front. Pharmacol. 2018, 9, 808 10.3389/fphar.2018.00808. 30104972 PMC6078047

[ref12] Ahlering M. T.; Yamamoto R. K., Elicker R. J.; Nguyen T. T.; Shulze J. E.; Zoethout J. J. Coating for Intraluminal Expandable Catheter Providing Contact Transfer of Drug Micro-Reservoirs. U.S. Patent 9,492,594 B2, Nov 15, 2016.

[ref13] Makadia H. K.; Siegel S. J. Poly Lactic-Co-Glycolic Acid (PLGA) as Biodegradable Controlled Drug Delivery Carrier. Polymers 2011, 3, 1377–1397. 10.3390/polym3031377. 22577513 PMC3347861

[ref14] Han F. Y.; Thurecht K. J.; Whittaker A. K.; Smith M. T. Bioerodable PLGA-Based Microparticles for Producing Sustained-Release Drug Formulations and Strategies for Improving Drug Loading. Front. Pharmacol. 2016, 7, 185 10.3389/fphar.2016.00185. 27445821 PMC4923250

[ref15] Park K.; Skidmore S.; Hadar J.; Garner J.; Park H.; Otte A.; Soh B. K.; Yoon G.; Yu D.; Yun Y.; Lee B. K.; Jiang X.; Wang Y. Injectable, Long-Acting PLGA Formulations: Analyzing PLGA and Understanding Microparticle Formation. J. Controlled Release 2019, 304, 125–134. 10.1016/j.jconrel.2019.05.003. 31071374

[ref16] Veldhuis G.; Gironès M.; Bingham D. Monodisperse Microspheres for Parenteral Drug Delivery. Drug Delivery Technol. 2009, 9, 24–31.

[ref17] Straub J. A.; Chickering D. E.; Church C. C.; Shah B.; Hanlon T.; Bernstein H. Porous PLGA Microparticles: AI-700, an Intravenously Administered Ultrasound Contrast Agent for Use in Echocardiography. J. Controlled Release 2005, 108, 21–32. 10.1016/j.jconrel.2005.07.020. 16126299

[ref18] Freitas S.; Merkle H. P.; Gander B. Microencapsulation by Solvent Extraction/Evaporation: Reviewing the State of the Art of Microsphere Preparation Process Technology. J. Controlled Release 2005, 102, 313–332. 10.1016/j.jconrel.2004.10.015. 15653154

[ref19] Ito F.; Makino K. Preparation and Properties of Monodispersed Rifampicin-Loaded Poly(Lactide-Co-Glycolide) Microspheres. Colloids Surf., B 2004, 39, 17–21. 10.1016/j.colsurfb.2004.08.016. 15542335

[ref20] Dragosavac M. M.; Vladisavljević G. T.; Holdich R. G.; Stillwell M. T. Production of Porous Silica Microparticles by Membrane Emulsification. Langmuir 2012, 28, 134–143. 10.1021/la202974b. 22059928

[ref21] Garstecki P.; Fuerstman M. J.; Stone H. A.; Whitesides G. M. Formation of Droplets and Bubbles in a Microfluidic T-Junction-Scaling and Mechanism of Break-Up. Lab Chip 2006, 6, 437–446. 10.1039/b510841a. 16511628

[ref22] Huang H.; He X. Fluid Displacement during Droplet Formation at Microfluidic Flow-Focusing Junctions. Lab Chip 2015, 15, 4197–4205. 10.1039/c5lc00730e. 26381220 PMC4605896

[ref23] Nabavi S. A.; Vladisavljević G. T.; Bandulasena M. V.; Arjmandi-Tash O.; Manović V. Prediction and Control of Drop Formation Modes in Microfluidic Generation of Double Emulsions by Single-Step Emulsification. J. Colloid Interface Sci. 2017, 505, 315–324. 10.1016/j.jcis.2017.05.115. 28601740

[ref24] Amstad E.; Chemama M.; Eggersdorfer M.; Arriaga L. R.; Brenner M. P.; Weitz D. A. Robust Scalable High Throughput Production of Monodisperse Drops. Lab Chip 2016, 16, 4163–4172. 10.1039/c6lc01075j. 27714028

[ref25] Li Z.; Leshansky A. M.; Pismen L. M.; Tabeling P. Step-Emulsification in a Microfluidic Device. Lab Chip 2015, 15, 1023–1031. 10.1039/c4lc01289e. 25490544

[ref26] Shi Z.; Lai X.; Sun C.; Zhang X.; Zhang L.; Pu Z.; Wang R.; Yu H.; Li D. Step Emulsification in Microfluidic Droplet Generation: Mechanisms and Structures. Chem. Commun. 2020, 56, 9056–9066. 10.1039/d0cc03628e. 32744276

[ref27] Kawakatsu T.; Kikuchi Y.; Nakajima M. Regular-Sized Cell Creation in Microchannel Emulsification by Visual Microprocessing Method. J. Am. Oil Chem. Soc. 1997, 74, 317–321. 10.1007/s11746-997-0143-8.

[ref28] Ofner A.; Moore D. G.; Rühs P. A.; Schwendimann P.; Eggersdorfer M.; Amstad E.; Weitz D. A.; Studart A. R. High-Throughput Step Emulsification for the Production of Functional Materials Using a Glass Microfluidic Device. Macromol. Chem. Phys. 2017, 218, 1600472 10.1002/macp.201600472.

[ref29] Opalski A. S.; Makuch K.; Lai Y.-K.; Derzsi L.; Garstecki P. Grooved Step Emulsification Systems Optimize the Throughput of Passive Generation of Monodisperse Emulsions. Lab Chip 2019, 19, 1183–1192. 10.1039/c8lc01096j. 30843018

[ref30] Kobayashi I.; Wada Y.; Uemura K.; Nakajima M. Microchannel Emulsification for Mass Production of Uniform Fine Droplets: Integration of Microchannel Arrays on a Chip. Microfluid. Nanofluid. 2010, 8, 255–262. 10.1007/s10404-009-0501-y.

[ref31] Vladisavljević G. T.; Ekanem E. E.; Zhang Z.; Khalid N.; Kobayashi I.; Nakajima M. Long-Term Stability of Droplet Production by Microchannel (Step) Emulsification in Microfluidic Silicon Chips with Large Number of Terraced Microchannels. Chem. Eng. J. 2018, 333, 380–391. 10.1016/j.cej.2017.09.141.

[ref32] Othman R.; Vladisavljević G. T.; Nagy Z. K.; Holdich R. G. Encapsulation and Controlled Release of Rapamycin from Polycaprolactone Nanoparticles Prepared by Membrane Micromixing Combined with Antisolvent Precipitation. Langmuir 2016, 32, 10685–10693. 10.1021/acs.langmuir.6b03178. 27690454

[ref33] Vladisavljević G. T.; Schubert H. Influence of Process Parameters on Droplet Size Distribution in SPG Membrane Emulsification and Stability of Prepared Emulsion Droplets. J. Membr. Sci. 2003, 225, 15–23. 10.1016/s0376-7388(03)00212-6.

[ref34] Kawakatsu T.; Trägårdh G.; Kikuchi Y.; Nakajima M.; Komori H.; Yonemoto T. Effect of Microchannel Structure on Droplet Size During Crossflow Microchannel Emulsification. J. Surfactants Deterg. 2000, 3, 295–302. 10.1007/s11743-000-0132-1.

[ref35] Schwendeman S. P.; Shah R. B.; Bailey B. A.; Schwendeman A. S. Injectable Controlled Release Depots for Large Molecules. J. Controlled Release 2014, 190, 240–253. 10.1016/j.jconrel.2014.05.057. PMC4261190 24929039

[ref36] Petersen H.; Bize J.-C.; Schuetz H.; Delporte M.-L. Pharmacokinetic and Technical Comparison of Sandostatin LAR and Other Formulations of Long-Acting Octreotide. BMC Res. Notes 2011, 4, 344 10.1186/1756-0500-4-344. 21906300 PMC3212992

[ref37] Petrova E. A.; Kedik S. A.; Alekseev K. V.; Blynskaya E. V.; Panov A. V.; Suslov V. V.; Tikhonova N. V. Influence of Microencapsulation Process Parameters on Naltrexone Prolonged-Release Dosage Form. Pharm. Chem. J. 2014, 48, 65–68. 10.1007/s11094-014-1048-0.

[ref38] Zhou J. Understanding Microencapsulation and Performance of CompositionEquivalent PLGA Microspheres for 1-Month Controlled Release of Leuprolide. Ph.D. Thesis, University of Michigan, 2019.

[ref39] Gill H. S.; Prausnitz M. R. Does Needle Size Matter?. J. Diabetes Sci. Technol. 2007, 1, 725–729. 10.1177/193229680700100517. 19885141 PMC2769648

[ref40] Barkley S.; Scarfe S. J.; Weeks E. R.; Dalnoki-Veress K. Predicting the Size of Droplets Produced Through Laplace Pressure Induced Snap-Off. Soft Matter 2016, 12, 7398–7404. 10.1039/c6sm00853d. 27535011

[ref41] Sugiura S.; Nakajima M.; Kumazawa N.; Iwamoto S.; Seki M. Characterization of Spontaneous Transformation-Based Droplet Formation during Microchannel Emulsification. J. Phys. Chem. B 2002, 106, 9405–9409. 10.1021/jp0259871.

[ref42] Mittal N.; Cohen C.; Bibette J.; Bremond N. Dynamics of Step-Emulsification: From a Single to a Collection of Emulsion Droplet Generators. Phys. Fluids 2014, 26, 082109 10.1063/1.4892949.

[ref43] Sahoo S. K.; Panyam J.; Prabha S.; Labhasetwar V. Residual Polyvinyl Alcohol Associated with Poly (D,L-Lactide-co-Glycolide) Nanoparticles Affects their Physical Properties and Cellular Uptake. J. Controlled Release 2002, 82, 105–114. 10.1016/s0168-3659(02)00127-x. 12106981

[ref44] Sackett D. L.; Wolff J. Nile Red as a Polarity-Sensitive Fluorescent Probe of Hydrophobic Protein Surfaces. Anal. Biochem. 1987, 167, 228–234. 10.1016/0003-2697(87)90157-6. 3442318

[ref45] Ekanem E. E.; Nabavi S. A.; Vladisavljević G. T.; Gu S. Structured Biodegradable Polymeric Microparticles for Drug Delivery Produced Using Flow Focusing Glass Microfluidic Devices. ACS Appl. Mater. Interfaces 2015, 7, 23132–23143. 10.1021/acsami.5b06943. 26423218

[ref46] Ekanem E. E.; Zhang Z.; Vladisavljević G. T. Facile Production of Biodegradable Bipolymer Patchy and Patchy Janus Particles with Controlled Morphology by Microfluidic Routes. Langmuir 2017, 33, 8476–8482. 10.1021/acs.langmuir.7b02506. 28776999

[ref47] Baek N.; Lee J.; Park K. Aqueous N,N-Diethylnicotinamide (DENA) Solution as a Medium for Accelerated Release Study of Paclitaxel. J. Biomater. Sci., Polym. Ed. 2004, 15, 527–542. 10.1163/156856204323005343. 15212332

[ref48] Elkharraz K.; Faisant N.; Guse C.; Siepmann F.; Arica-Yegin B.; Oger J. M.; Gust R.; Goepferich A.; Benoit J. P.; Siepmann J. Paclitaxel-Loaded Microparticles and Implants for the Treatment of Brain Cancer: Preparation and Physicochemical Characterization. Int. J. Pharm. 2006, 18, 127–136. 10.1016/j.ijpharm.2005.07.028. 16490330

[ref49] Booth J. J.; Omar M.; Abbott S.; Shimizu S. Hydrotrope Accumulation around the Drug: The Driving Force for Solubilization and Minimum Hydrotrope Concentration for Nicotinamide and Urea. Phys. Chem. Chem. Phys. 2015, 17, 8028–8037. 10.1039/c4cp05414h. 25723588

[ref50] Ridolfo R.; Tavakoli S.; Junnuthula V.; Williams D. S.; Urtti A.; van Hest J. C. M. Exploring the Impact of Morphology on the Properties of Biodegradable Nanoparticles and Their Diffusion in Complex Biological Medium. Biomacromolecules 2021, 22, 126–133. 10.1021/acs.biomac.0c00726. 32510218 PMC7805011

[ref51] Yoo J.; Won Y.-Y. Phenomenology of the Initial Burst Release of Drugs from PLGA Microparticles. ACS Biomater. Sci. Eng. 2020, 6, 6053–6062. 10.1021/acsbiomaterials.0c01228. 33449671

