# Supplementary material for: Monodispersed Sirolimus-Loaded PLGA Microspheres with a Controlled Degree of Drug–Polymer Phase Separation for Drug-Coated Implantable Medical Devices and Subcutaneous Injection
Source: ACS Appl Bio Mater. 2022 Jul 16;5(8):3766–77. doi: 10.1021/acsabm.2c00319 (PMC9382632; doi:10.1021/acsabm.2c00319)
Supplement: Supplementary file 1 — mt2c00319_si_001.pdf [file mt2c00319_si_001.pdf]

Supporting information

Monodispersed sirolimus loaded PLGA microspheres with controlled degree of drug-polymer phase separation for drug-coated implantable medical devices and subcutaneous injection

Zilin Zhang,[a,b] Ekanem E. Ekanem,[a,c] Mitsutoshi Nakajima,[d] Guido Bolognesi,[a] and Goran T. Vladisavljević[a]*
[a]Department of Chemical Engineering, Loughborough University, Loughborough LE11 3TU, United Kingdom.
[b]Guangxi Key Laboratory of Green Chemical Materials and Safety Technology, Beibu Gulf University, Qinzhou 535011, China
[c]Department of Chemical Engineering, University of Bath, Bath BA2 7AY, United Kingdom.
[d]Faculty of Life and Environmental Sciences, University of Tsukuba, 1-1-1 Tennoudai, Tsukuba, Ibaraki 305-8572, Japan.

Supporting information S1

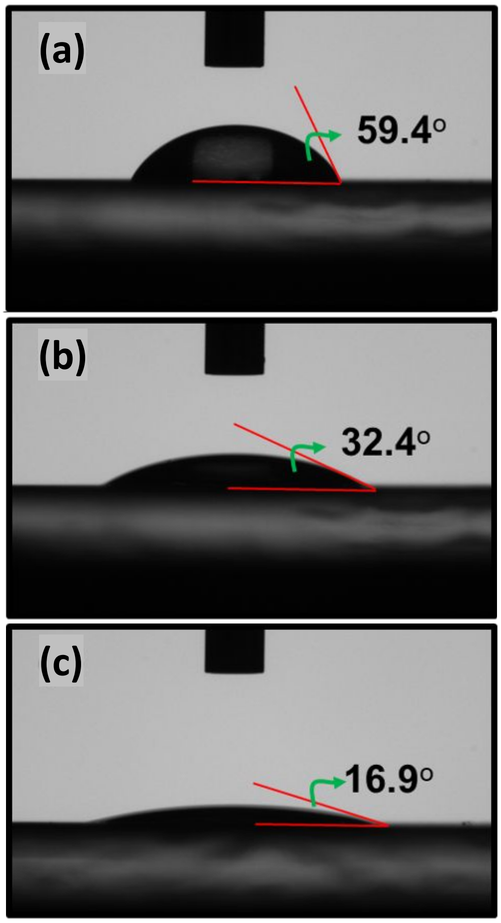

Figure S1. Contact angle between pure water and a chip surface: (a) contaminated surface before cleaning; (b) surface after washing with DCM; (c) surface after plasma oxidation.

*Corresponding author's email: G.Vladisavljevic@lboroac.uk.

Supporting information S2

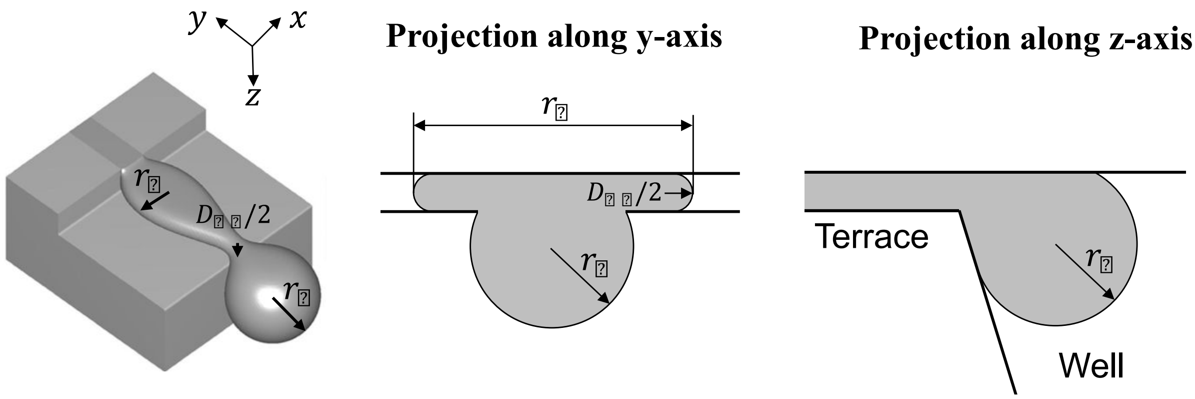

Figure S2. Droplet formation by step microfluidic emulsification using a terraced microchannel. The channel and terrace depth is D_{MC}.

Supporting information S3

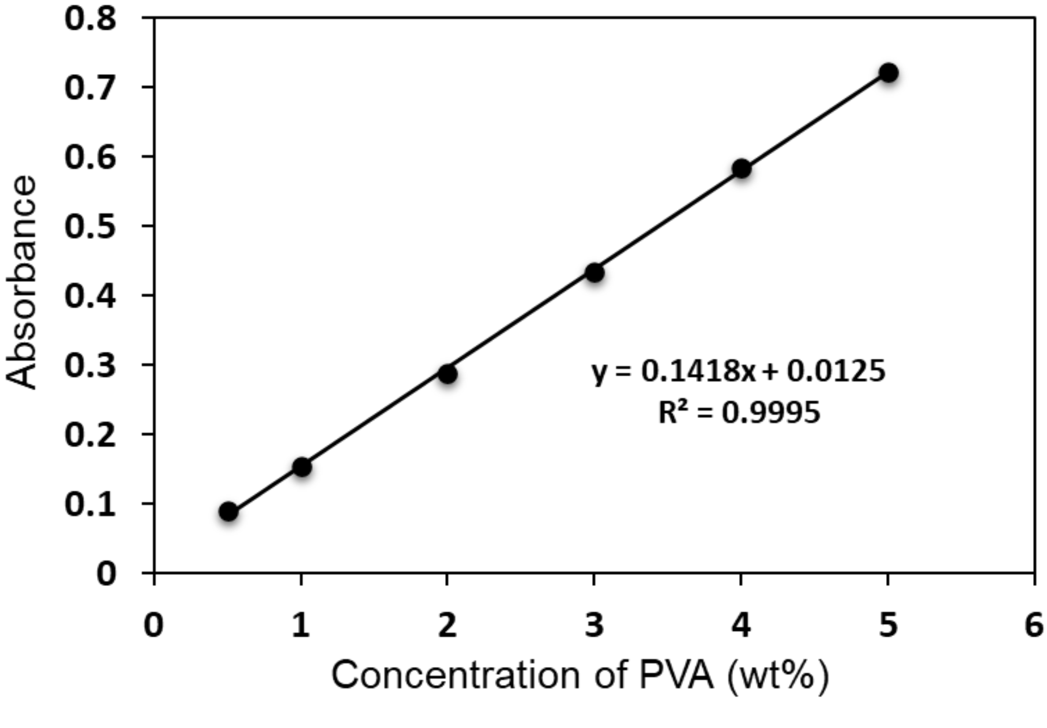

Figure S3. Calibration curve of PVA for UV-vis analysis.

Supporting information S4

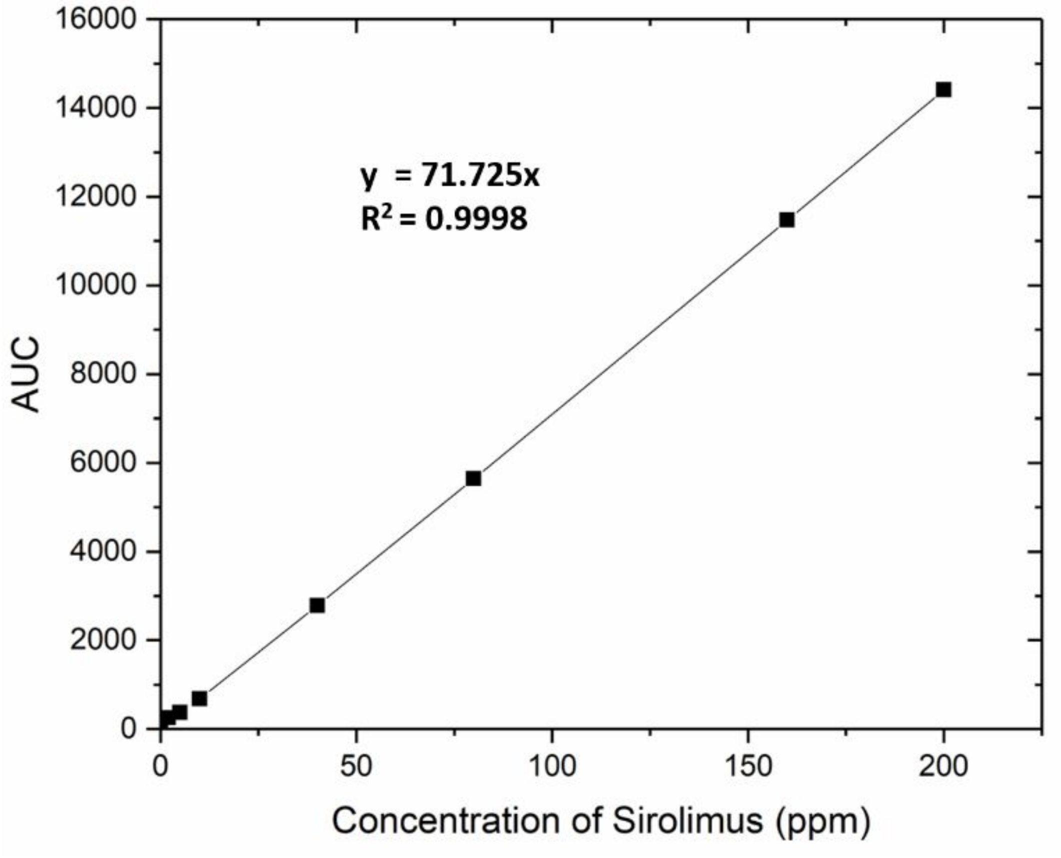

Figure S4. Calibration curve of sirolimus for HPLC analysis.
